# Case Report: The Genetic Diagnosis of Duchenne Muscular Dystrophy in the Middle East

**DOI:** 10.3389/fped.2021.716424

**Published:** 2021-09-13

**Authors:** Fouad Alghamdi, Asmaa Al-Tawari, Hadil Alrohaif, Walaa Alshuaibi, Hicham Mansour, Annemieke Aartsma-Rus, André Mégarbané

**Affiliations:** ^1^Neuroscience Center, King Fahad Specialist Hospital, Dammam, Saudi Arabia; ^2^Pediatric Neurology Unit, Pediatric Department, Al-Sabah Hospital, Kuwait City, Kuwait; ^3^Kuwait Medical Genetics Centre, Al-Sabah Hospital, Kuwait City, Kuwait; ^4^College of Medicine, King Saud University, Riyadh, Saudi Arabia; ^5^Medical Genetics Division, Pediatrics Department, King Saud University, Riyadh, Saudi Arabia; ^6^Pediatric Department, Saint George Hospital, Balamand University, Beirut, Lebanon; ^7^Department of Human Genetics, Leiden University Medical Center, Leiden, Netherlands; ^8^Department of Human Genetics, Gilbert and Rose-Marie Chagoury School of Medicine, Lebanese American University, Byblos, Lebanon

**Keywords:** case report, diagnostic delay, DMD, genetic diagnosis, neuromuscular disorder

## Abstract

The timely and accurate genetic diagnosis of Duchenne muscular dystrophy (DMD) enables prompt initiation of disease management and genetic counseling and optimal patient care. Despite the existence of best practice guidelines for the diagnosis of DMD, implementation of these recommendations in different parts of the world is challenging. Here, we present 4 unique case studies which illustrate the different diagnostic pathways of patients with DMD in Middle Eastern countries and highlight region-specific challenges to achieving timely and accurate genetic diagnosis of DMD. A lack of disease awareness and consequential failure to recognize the signs and symptoms of DMD significantly contributed to the delayed diagnoses of these patients. Additional challenges included limited available funding for genetic testing and a lack of local specialist and genetic testing centers, causing patients and their families to travel vast distances for appointments in some countries. Earlier and more accurate genetic diagnosis of DMD in this region would allow patients to benefit from effective disease management, leading to improvements in health-related quality of life.

## Introduction

Duchenne muscular dystrophy (DMD) is a rare, X-linked neuromuscular disorder affecting ~1 in every 3,600–6,000 live male births (1–3). Patients experience progressive muscle weakness and motor function decline (1). DMD is caused by large deletions/duplications or small mutations in the *DMD* gene, preventing functional dystrophin protein production (1, 4).

A timely, accurate genetic diagnosis is crucial for optimal patient care, including early initiation of disease management which slows functional decline. Early and accurate diagnosis is also important for informing on eligibility for mutation-specific therapies, and identifying female carriers of DMD in the family (1, 4). Best practice guidelines for diagnosing DMD were developed by the DMD Care Considerations Working Group based on standard of care considerations (1). When signs and symptoms or developmental delays are noticed, or when serum creatine kinase (CK) concentrations are increased, the guidelines recommend referral to a neuromuscular specialist for genetic testing. Given that ~70% of patients have a single- or multi-exon deletion/duplication, it is cost effective to screen for these mutations first, using multiplex ligation-dependent probe amplification (MLPA) or array comparative genomic hybridization (4). In ~25–30% of cases, a mutation is not found and genetic sequencing is required to detect small mutations (4). If no diagnosis has been reached after these steps, a muscle biopsy should be taken to analyze dystrophin protein levels and/or mRNA transcripts (1, 4).

Most female carriers of DMD are asymptomatic: 50% of their muscle fibers are positive for dystrophin owing to random X-chromosome inactivation (4, 5). Over time, this percentage can increase due to a survival advantage of dystrophin-positive fibers (6). However, in rare cases, DMD disease can occur in female individuals, sometimes via a translocation involving the *DMD* gene on the X chromosome (4). X-inactivation occurs early in embryogenesis and daughter cells inherit the pattern of inactivation (7). Cells in which the translocated X chromosome is inactivated do not survive. Therefore, the embryo will only contain cells in which the non-translocated X chromosome with the unaffected *DMD* gene is inactivated, so no dystrophin is produced (4).

**Figure 1:**
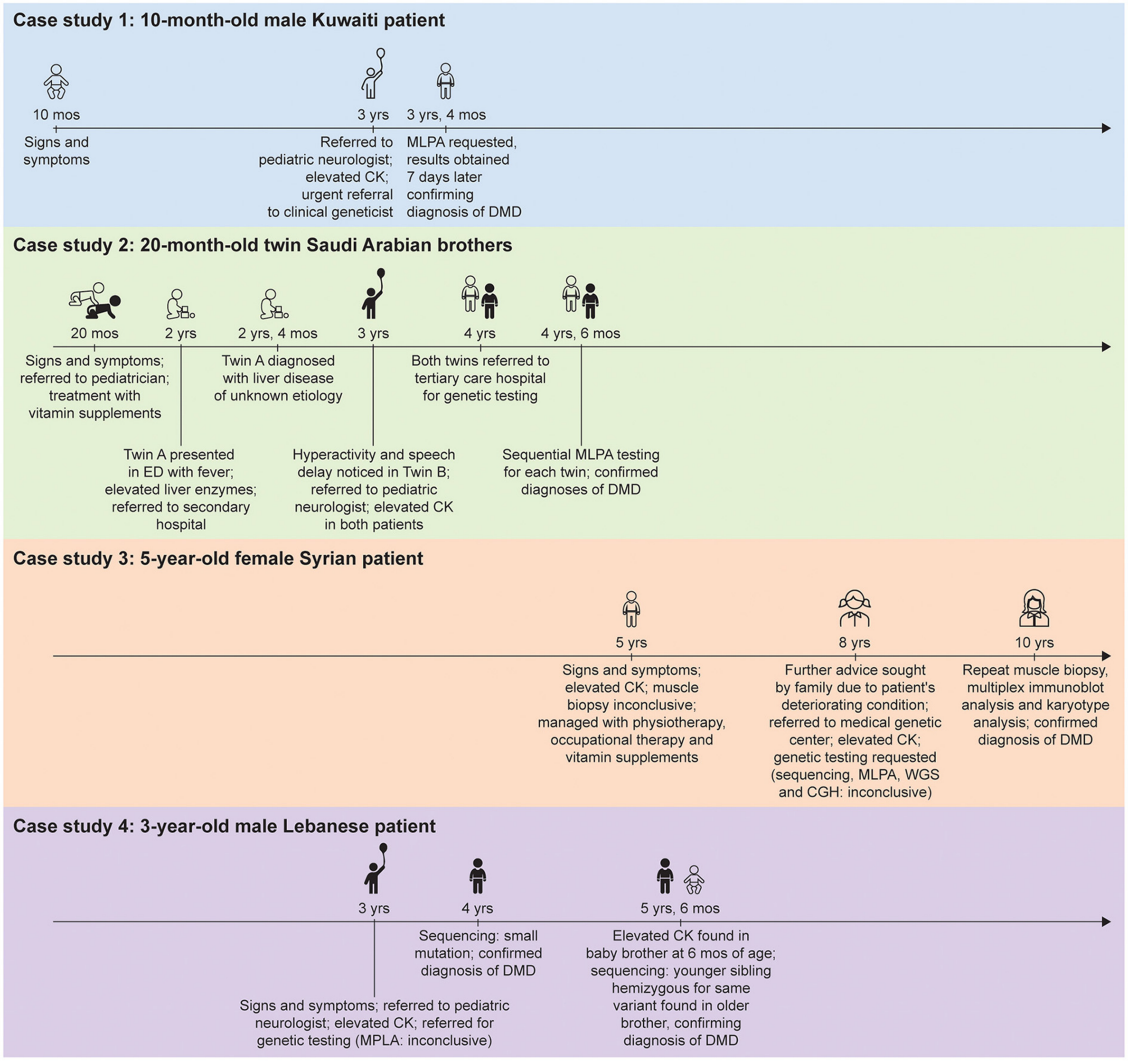
Timeline from presentation of first signs and symptoms of disease to genetic diagnosis for the patients described in the case studies. CGH, comparative genomic hybridization; CK, creatine kinase; DMD, Duchenne muscular dystrophy; ED, emergency department; MLPA, multiplex ligation-dependent probe amplification; WGS, whole genome sequencing.

Implementation of best practice across the world, including in the Middle East, can be challenging owing to logistical and cultural barriers (8). The lack of standardized DMD referral and diagnostic pathways in the Middle East is a significant hurdle to timely diagnosis (8). Published studies of DMD diagnosis and regional-specific advice on the implementation of best practice in the Middle East are also lacking. We present 4 case studies of the genetic diagnostic pathway of patients with DMD in the Middle East, each described by a pediatric neurologist and a clinical geneticist (Figure 1). We highlight the challenges and discuss ways to improve genetic diagnosis of DMD in this region.

## Case Description

### Case Description 1

A 10-month-old male Kuwaiti patient with a family history of DMD presented with hypotonia and mild motor milestone delay to the primary care physician (PCP). At 3 years of age, the patient was referred to a pediatric neurologist at a nearby specialist medical center by the PCP. According to established best practice in Kuwait, a CK test was immediately conducted, showing elevated levels of >24,000 U/L.

MLPA analysis was promptly requested by a clinical geneticist at the local genetics center, following an urgent referral from the pediatric neurologist. The test was reimbursed by the Kuwaiti government, and the results were obtained 7 days later. A deletion of exons 46–49 in the *DMD* gene was observed, confirming a diagnosis of DMD when the patient was 3 years and 4 months old. The clinical geneticist wrote the genetic test report and communicated the diagnosis to the patient's family, who were referred for genetic counseling at the local hospital.

### Case Description 2

Twin brothers from rural southern Saudi Arabia with no family history of DMD both presented with delayed walking and frequent falling at ~20 months of age. The parents consulted a pediatrician, who suggested a vitamin deficiency and started treatment with vitamin D3 supplements.

At 2 years of age, twin A presented in an emergency department with a fever, and routine tests revealed elevated liver enzyme concentrations, which were attributed to a viral infection. However, liver enzyme concentrations remained elevated several weeks later, so the patient was referred to a secondary hospital 4 h away. The twins were ~2 years and 4 months old when twin A was diagnosed with a liver disease of unknown etiology following inconclusive results of a liver biopsy and extensive tests examining infectious, metabolic, and autoimmune etiologies.

At ~3 years of age, the family reported hyperactivity and a speech delay in twin B. The patient was referred to a pediatric neurologist, who noticed muscle weakness, depressed deep tendon reflexes, and Gowers' signs in both boys upon assessment ~6 months later. Blood tests showed CK levels of >22,000 U/L in both patients. DMD was suspected so both patients, now ~4 years old, were referred to a tertiary care hospital 10 h away for genetic testing.

MLPA testing was performed in collaboration with a company in Spain and reimbursed by the Saudi Arabian government. Initially, MLPA testing was requested for one patient only. Once the results were obtained 3 months later, showing a hemizygous duplication of exon 12 of the *DMD* gene and thereby confirming the diagnosis of DMD, MLPA testing for the second twin was requested. The second set of results obtained another month later showed the same mutation and confirmed the second diagnosis, by which time the twins were 4 years, 6 months old.

### Case Description 3

A 5-year-old female Syrian patient with no family history of DMD presented to the PCP with walking problems and recurrent falls. Prompted by clinical features suggestive of a neuromuscular disorder, the PCP requested a CK analysis, which demonstrated levels of >10,000 U/L. Although genetic testing is recommended upon finding elevated CK levels (1), a muscle biopsy was performed instead. The results were suggestive of a muscular dystrophy, but a lack of resources and expertise prevented a conclusive demonstration of depleted dystrophin levels. The patient was managed with physiotherapy, occupational therapy, and vitamin supplements.

By the time the patient was 8 years old, her family had noticed increasing fatigue, enlarged calves, and frequent falls. The PCP referred the patient to a clinical geneticist at a Lebanese medical genetic center ~120 km away. Upon examination, the patient had mild psychomotor delay, intellectual disability, hyperlordosis, and muscle weakness. Even at this stage, DMD was not suspected because of the rarity of disease in female individuals. Another blood test revealed CK levels of ~8,000 U/L, prompting a series of genetic tests. For each test, the family had to complete an ~240 km roundtrip. Because there were no Lebanese or Syrian government reimbursement schemes available, the patient's family paid for the genetic tests themselves, leading to delays in the transfer of payments and ultimately obtaining the results.

Gene sequencing and MLPA analysis of 123 neuromuscular genes, outsourced to the USA, provided inconclusive results regarding a diagnosis but did demonstrate the presence of variants of unknown significance. The parents were carriers of these variants, warranting an in-depth discussion between the clinical geneticist and the family. Four months later, array comparative genomic hybridization analysis was carried out, outsourced to Germany, to detect any large genomic deletions/duplications missed by gene sequencing, but no mutations were found. Lastly, when the patient was ~9 years old, whole genome sequencing was performed, also in Germany, and again provided inconclusive results regarding a diagnosis.

A second muscle biopsy was performed when the patient was almost 10 years old. Multiplex immunoblot analysis unequivocally showed the absence of dystrophin protein. A karyotype analysis then showed the presence of a *de novo* X-chromosome translocation near the site of the *DMD* gene (t[X;1][p21.3;p22.2]), confirming a DMD diagnosis in the female patient. Owing to the complexity of the genetic test results, further discussions between the clinical geneticist and the family were required to explain the results and the final diagnosis.

### Case Description 4

A 3-year-old male patient based in rural Lebanon with no family history of DMD presented to the PCP with enlarged calf muscles and fatigue. Prompt referral to a pediatric neurologist who requested a blood test revealed elevated CK levels (>12,000 U/L). The patient, suspected to have DMD, was referred for genetic testing; however, MLPA analysis failed to detect a mutation. MLPA analysis uses probes to amplify specific segments of DNA to detect exon deletions or duplications and as such is unable to detect unknown point mutations (9). As a result, the clinical geneticist sought the support of a French laboratory for genetic sequencing as the family could not afford the test. It took ~4/5 months to obtain the genetic test results, which revealed a small point mutation in the *DMD* gene (c.9249G>A p.[Trp3083^*^]), confirming a diagnosis of DMD by which stage the patient was ~4 years old. Although the mother did not carry the same mutation, the genetic test report, highlighted a potential risk of DMD in future children due to gonadal mosaicism. Despite this being explained to the family by the clinical geneticist, there was some doubt as to whether the family understood the implications of the genetic test results for family planning.

Two years later, the mother delivered a second boy, who at 6 months of age was found to have elevated CK following the parents' request for CK analysis. The family contacted the clinical geneticist who diagnosed the brother with DMD. Sequencing revealed that the second child was hemizygous for the same variant found in his older brother, confirming a diagnosis of DMD.

## Discussion

A lack of disease awareness and subsequent failure to recognize DMD signs and symptoms, especially atypical presentations of disease, leads to delayed diagnosis of DMD worldwide (10, 11). For the Saudi Arabian twins, a lack of DMD awareness resulted in misdiagnoses, late referral to the tertiary care hospital, and delayed diagnoses at 4 years, 6 months of age. A delay in the diagnosis of the Syrian patient was similarly attributed at least in part to the lack of suspicion of DMD. In this case, the rarity of female DMD added further complexity. Furthermore, it was challenging to explain the extremely complex genetic test results to this patient's family, highlighting the need to increase education on DMD as well as disease awareness among the public. Even in the case of the Kuwaiti patient, for whom a relatively quick diagnosis from initial signs and symptoms was achieved, the initial delay between first signs and symptoms and referral to a pediatric neurologist was attributed to a lack of caregiver and primary care disease awareness. Direct referral from primary care to the genetic center could also have saved time to diagnosis in this case. Disease awareness campaigns about DMD and the diagnostic care guidelines could profoundly reduce the time for referral and future diagnostic delays in the Middle East and globally (8).

A prominent logistical challenge was the vast geographical distances families were required to travel to reach specialist centers. In large countries such as Saudi Arabia, where only the tertiary hospitals have the genetic facilities to confirm a diagnosis, this challenge is particularly pertinent. Referral of the Syrian patient to a specialist medical genetics center involved traveling ~120 km each way per visit. Conversely, the availability of local genetic testing facilities was one reason why there was little delay in diagnosing the Kuwaiti patient.

Funding deficits represent a further barrier to genetic diagnosis, often driven by a lack of governmental reimbursement schemes. The Lebanese patient's family could not afford gene sequencing, prompting outsourcing to a laboratory abroad and delaying the obtainment of confirmative genetic test results. The family of the Syrian patient had to pay for genetic testing, leading to further diagnostic delays. A lack of specific resources and expertise was also the reason for an inconclusive result of the initial muscle biopsy in this patient. Notably, the care guidelines indicate that a biopsy should be used as a last resort to diagnose DMD. In >99% of cases the genetic mutation can be detected on DNA level and an invasive muscle biopsy is not required. By contrast, when a biopsy reveals absence of dystrophin, DNA analysis to identify the genetic mutation, which is crucial for genetic counseling, is still required.

The standard of care guidelines also recommend a multidisciplinary approach to the management of patients with DMD, including giving corticosteroids, the benefits of which have been extensively demonstrated (1, 12). Soon after diagnosis, all patients started on corticosteroids. Although the Saudi twins remain on corticosteroid therapy now, the family were hesitant to continue treatment owing to the observed weight gain in the boys, a known side effect. Indeed, the family of the Syrian patient stopped the treatment after 6 months as they believed that corticosteroid-induced weight gain was contributing to walking difficulties in their daughter. The twins' functional ability also continued to decline despite treatment, causing the family to question the importance of therapy. Raising disease awareness will also increase knowledge of available treatment options, helping to address any stigma associated with corticosteroid treatment and facilitating more effective and timely therapeutic management of patients.

Whilst this report is limited to a description of only 4 individual case studies, it provides a foundation and highlights the need for future studies on the genetic diagnosis of DMD in the region. The patients described in each of the case studies presented here harbored distinct genetic mutations within the *DMD* gene, meaning different diagnostic pathways were undertaken to achieve confirmed diagnoses. Therefore, a strength of our report is that it highlights the various diagnostic challenges encountered in Middle Eastern countries for patients with different *DMD* mutations. Furthermore, our case report demonstrates that many of these diagnostic challenges are common amongst patients with DMD, regardless of the specific genetic mutation. Each case study was described by both a pediatric neurologist and clinical geneticist, providing a complete clinical perspective on the diagnostic journey undertaken by each patient. Some region-specific challenges are also highlighted here, given the different geographical locations of each case study.

Here, we present case studies illustrating different genetic diagnostic pathways of patients with DMD that highlight region-specific challenges to achieving a timely and accurate genetic diagnosis in Middle Eastern countries. Earlier, accurate diagnosis of DMD would allow patients to benefit from more effective management and treatment, leading to improved health-related quality of life for them and their families. Future initiatives should focus not only on increasing disease awareness, but also on providing support for the regional implementation of best practice for diagnosis.

## Data Availability Statement

The original contributions presented in the study are included in the article/Supplementary Material, further inquiries can be directed to the corresponding authors.

## Ethics Statement

Ethical review and approval was not required for the study on human participants in accordance with the local legislation and institutional requirements. Written informed consent to participate in this study was provided by the participants' legal guardian/next of kin.

## Author Contributions

All authors contributed to the article, revised all drafts and approved the submitted version.

## Funding

This work was funded by PTC Therapeutics and GenPharm.

## Conflict of Interest

FA has received honoraria for consulting, advisory meetings and speaker activities from AveXis, Biogen, Biologix, Genpharm, PTC Therapeutics, Roche, Sanofi Genzyme and Sarepta Therapeutics. AA-R is employed by Leiden University Medical Center (LUMC), which has patents on exon skipping technology and as a co-inventor, AA-R is entitled to a share of the royalties. AA-R has also acted as a consultant for Alpha Anomeric, AstraZeneca, BioMarin, CRISPR Therapeutics, Deerfield, Eisai, GLC Consulting, Grünenthal, Guidepoint Global, PTC Therapeutics, Sarepta Therapeutics, Summit Therapeutics, and Wave Therapeutics (all remunerations go to LUMC) and participated as a scientific advisory board member of Hybridize Therapeutics, Philae Pharmaceuticals, ProQR Therapeutics, Sarepta Therapeutics and Silence Therapeutics (all remunerations go to LUMC). The remaining authors declare that the research was conducted in the absence of any commercial or financial relationships that could be construed as a potential conflict of interest. The authors declare that this study received funding from PTC Therapeutics and GenPharm. PTC Therapeutics and GenPharm had no involvement in the study design, the collection, analysis and interpretation of data, or the writing of the report. The concept for the manuscript was agreed between PTC Therapeutics and the authors at a Roundtable meeting funded by PTC Therapeutics and the decision to submit the manuscript for publication was made by PTC Therapeutics and the authors.

## Publisher's Note

All claims expressed in this article are solely those of the authors and do not necessarily represent those of their affiliated organizations, or those of the publisher, the editors and the reviewers. Any product that may be evaluated in this article, or claim that may be made by its manufacturer, is not guaranteed or endorsed by the publisher.

## Abbreviations

CK: creatine kinase
DMD: Duchenne muscular dystrophy
MLPA: multiplex ligation-dependent probe amplification
PCP: primary care physician.
